# Fostering Mental Health Literacy Among Primary School Professionals: Evaluating the Impact of an Online Training Program ‘Well@School’

**DOI:** 10.3390/ijerph22030435

**Published:** 2025-03-15

**Authors:** Joonas Korhonen, Mari Lahti, Kostadin Kostadinov, Karmen Erjavec, Natalja Istomina, Svetla Ivanova, Areti Lagiou, Valentina Lalova, Monika Makutienė, Venetia Notara, Hanna Ollikkala, Gergana Petrova, Evanthia Sakellari, Daiva Sukyte, Camilla Laaksonen

**Affiliations:** 1 Department of Nursing Science, Faculty of Medicine, University of Turku, 20014 Turku, Finland; mari.lahti@turkuamk.fi; 2 Emergency Care, Public Health Nursing, Midwifery and Diagnostic Services, Faculty of Health and Well-Being, Turku University of Applied Science, 20520 Turku, Finland; hanna.ollikkala@turkuamk.fi (H.O.); camilla.laaksonen@turkuamk.fi (C.L.); 3 Department of Social Medicine and Public Health, Faculty of Public Health, Medical University of Plovdiv, 4002 Plovdiv, Bulgaria; kostadinr.kostadinov@mu-plovdiv.bg; 4 Environmental Health Division, Research Institute at Medical University of Plovdiv, 15-A “Vasil Aprilov” Blvd., 4002 Plovdiv, Bulgaria; 5 Faculty of Health Sciences, University of Novo mesto, 8000 Novo mesto, Slovenia; karmen.erjavec@uni-nm.si; 6 Institute of Health Science, Faculty of Medicine, Vilnius University, 01513 Vilnius, Lithuania; natalja.istomina@mf.vu.lt (N.I.); monika.makutiene@mf.vu.lt (M.M.); dr.daiva.sukyte@gmail.com (D.S.); 7 Department of Nursing Care, Faculty of Public Health, Medical University of Plovdiv, 4002 Plovdiv, Bulgaria; svetla.ivanova@mu-plovdiv.bg (S.I.); valentina.lalova@mu-plovdiv.bg (V.L.); gergana.petrova@mu-plovdiv.bg (G.P.); 8 Laboratory of Hygiene and Epidemiology, Department of Public and Community Health, School of Public Health, University of West Attica, 12210 Athens, Greece; alagiou@uniwa.gr (A.L.); vnotara@uniwa.gr (V.N.); sakellari@uniwa.gr (E.S.)

**Keywords:** child and adolescent, digital health, health literacy, mental health promotion, public health

## Abstract

Mental health challenges among children and adolescents have become a pressing global concern, particularly in the wake of the COVID-19 pandemic and ongoing geopolitical instability. Addressing these issues requires innovative, cost-effective strategies, with schools serving as critical platforms for mental health promotion. This study evaluates the impact of an online training program, Well@School, designed to enhance Mental Health Literacy (MHL) among primary school professionals in Finland, Lithuania, Bulgaria, Slovenia, and Greece. Using a descriptive, cross-sectional design with pre- and post-test assessments, the study involved 223 health, education, and social care professionals. The revised Mental Health Literacy Scale (MHLS) was employed to measure changes in MHL. Results indicated a significant positive effect, with an average increase of 4 points (2.5%) in MHLS scores post-course. Bayesian analysis further confirmed this improvement, showing a high probability (99.92%) of a positive impact, with the most likely gain ranging between 3 and 5 points. The findings underscore the potential of online training programs to enhance MHL among school professionals, thereby improving their capacity to support students’ mental health. This study highlights the importance of equipping primary school staff with the necessary skills to recognize and address mental health challenges, reduce stigma, and foster a supportive school environment.

## 1. Introduction

Mental health challenges among children and young people represent a significant global burden and remain a key priority within the framework of the Sustainable Development Goals (SDGs) [1,2]. The mental well-being of this demographic is increasingly at risk, not only due to the lingering consequences of the COVID-19 pandemic but also as a result of the unstable security situation in Europe and other regions worldwide [3,4]. These overlapping crises have exacerbated stressors such as social isolation, fear of illness, disruptions to education, and economic uncertainty within families, all of which can contribute to long-term psychological distress among youth [4].

In response to these challenges, innovative and cost-effective strategies have been explored to support mental health promotion, with a particular focus on integrating mental health education into school curricula. By enhancing public awareness and improving competencies related to emotional resilience, coping mechanisms, and mental health literacy, such initiatives aim to equip young individuals with essential life skills [5,6,7]. Schools are uniquely positioned to play a pivotal role in mental health promotion, as they provide a structured and accessible environment where large populations of children and adolescents can be reached. Moreover, schools serve as key platforms for early identification of mental health issues and the implementation of supportive interventions tailored to diverse needs.

However, the psychological impact of recent global events has placed unprecedented pressure on children and young people, highlighting the need for strengthened mental health support systems within educational settings. Given their central role in children’s daily lives, educational institutions serve as optimal settings for fostering well-being, providing not only academic instruction but also emotional and psychological support [8]. In this regard, school professionals, including teachers, counsellors, and support staff—are vital contributors to promoting mental health, ensuring that students receive the necessary guidance and resources to effectively navigate these challenges.

### 1.1. Multiprofessional Mental Health Promotion

Educators and other school staff serve as front-line advocates for promoting positive mental health, preventing mental health problems, and effectively identifying and addressing mental health challenges among children and young people [9]. Their role extends beyond academic instruction, encompassing emotional support, early intervention, and the creation of a psychologically safe learning environment. Additionally, school professionals play a crucial role in combating stigma and fostering a supportive and inclusive attitude toward individuals facing mental health-related difficulties, helping to normalize discussions about mental well-being and encourage help-seeking behaviors among students [10].

However, there are notable country-specific differences in the educational opportunities and training available to teachers, school health professionals, social care specialists, school managers, and other staff working in primary school environments [11]. These variations can impact the effectiveness of mental health promotion strategies, as well as the capacity of school professionals to respond to emerging mental health concerns. A recent report by the European Commission [12] highlights the increasing demands placed on educators, including heavier workloads, heightened stress levels, and concerns about educational equity. These pressures, combined with limited access to specialized mental health training, can affect the ability of school professionals to adequately support students’ well-being.

Given these challenges, a multiprofessional approach to mental health promotion in schools is essential. Collaboration between educators, healthcare professionals, social workers, and community organizations can enhance the effectiveness of mental health initiatives, ensuring that students receive comprehensive and coordinated support. Schools should be equipped with adequate resources and training programs that empower staff to recognize early signs of distress, implement preventive measures, and facilitate access to appropriate mental health services when needed.

### 1.2. Mental Health Literacy in Primary School

Mental health literacy (MHL) encompasses a set of essential skills and knowledge that promote mental well-being by fostering the recognition of mental health challenges, improving knowledge about mental disorders, enhancing help-seeking behaviors, and reducing stigma [13]. Encouraging overall health literacy—of which MHL is a key component—has been identified as a critical factor for achieving greater success in society, as it empowers individuals to make informed decisions about their health and well-being [14].

Recognizing the importance of MHL, various initiatives have been developed to integrate mental health education into school curricula. These initiatives aim not only to enhance students’ understanding of mental health but also to equip school professionals with the necessary competencies to support and guide students effectively. Existing school-based MHL programs have demonstrated positive outcomes, contributing to improved mental health awareness, reduced stigma, and better coping strategies among students and educators alike [10].

Developing training programs specifically for primary school professionals could be a valuable strategy in fostering MHL. Such initiatives can help reduce negative attitudes towards mental health issues and enhance educators’ ability to identify and support students facing mental health challenges [1,7,9]. Previous studies have highlighted key competencies required for promoting mental health in primary schools. These include a strong understanding of child development and age-specific mental health concerns, effective communication and intervention skills for supporting children, and a broader perception of educators as active health promoters within the school setting [15].

Additionally, as digital technologies become an integral part of children’s lives, there is a growing need for digital literacy training in the context of MHL. Research has identified gaps in digital mental health resources for primary schools, as well as a need for training programs that equip school professionals with the skills to implement digital tools effectively in mental health education [6,11]. Children and young people often turn to peers and family members rather than seeking professional help in times of distress, making it essential for schools to foster digital literacy that supports mental health learning. However, despite the increasing reliance on digital solutions in education, school-based mental health interventions utilizing digital methods remain scarce [6]. Further research is needed to assess the effectiveness of teacher training programs in enhancing MHL and to explore innovative approaches for integrating digital tools into primary school settings.

### 1.3. Well@School

This study is part of an Erasmus+ Strategic Partnership funded project Well@School (https://wellatschool.turkuamk.fi/, accessed on 9 March 2025). The international multi-center initiative aimed to enhance the competencies of primary school professionals to promote mental health within school communities through the use of digital methods. The project was implemented across five European countries—Finland, Lithuania, Bulgaria, Slovenia, and Greece—during the academic year 2022–2023. In this paper, we report on the findings from an online course designed to improve mental health literacy (MHL) among health, education, and social care professionals working in primary school settings. The focus of this paper is to present the impact of the Well@School online course on the MHL of the project’s target group.

The aim of this study is to assess the impact of an online course on MHL among professionals working in health, education, and social care in primary school settings. The objectives of the study were to explore the educational and training backgrounds of the school professionals participating in the online course, to examine the MHL outcomes achieved by these professionals, and to evaluate the overall influence of the course on improving their MHL.

## 2. Materials and Methods

### 2.1. Design and Setting

This study employed a descriptive, cross-sectional design with pre- and post-test evaluations [16] to assess the effectiveness of an online pilot course on Mental Health Literacy (MHL). The course was developed under the Well@School project and delivered via the Moodle platform across five European countries: Finland, Greece, Bulgaria, Slovenia, and Lithuania. The pre-test evaluation was conducted prior to the course to establish baseline MHL levels, while the post-test evaluation was administered immediately after course completion to measure changes in MHL.

### 2.2. Study Population Ad Sampling

The study population consisted of professionals in health, education, and social care working in primary school settings across the partner countries. Eligibility criteria included the following: (1) Being a professional in health, education, or social care; (2) Working in a primary school setting in one of the partner countries. (3) Willingness and ability to complete the online course and survey in English.

A convenience sampling approach was used to recruit participants who were readily available and willing to participate. An invitation to the online survey was sent to all 223 participants who enrolled in the pilot course. The number of participants in the pilot course was predefined in the project description, reflecting the logistical and resource constraints of the study. This sampling method was chosen due to the practical challenges of reaching professionals across multiple countries and ensuring timely participation within the project timeline. While convenience sampling may limit the generalizability of the findings, it was deemed the most feasible approach given the study’s cross-national scope and the need to align with the project’s predefined framework.

### 2.3. Data Collection

Data collection was conducted through an online survey administered over a six-month period, from October 2022 to March 2023. The process involved three key phases:

1. Pre-course survey: Administered from October 2022 to January 2023.
2. Pilot online course: Participants engaged in the online course, which was delivered via the Moodle platform.
3. Post-course survey: Conducted from January to March 2023, this survey assessed changes in MHL following course completion.

### 2.4. Mental Health Literacy Scale Instrument

The study utilized a revised version of the Mental Health Literacy Scale (MHLS) [17], a standardized tool originally developed by O’Connor and Casey (2015) [18] in the Australian context. The MHLS has been widely used and validated across multiple studies, demonstrating strong reliability (Cronbach’s α > 0.80) and validity in measuring MHL consistently and accurately [18,19,20].

The revised version of the MHLS [17,20,21] was selected for its adaptability to diverse cultural contexts, including low- and middle-income countries, where it has shown good psychometric performance. This version aligns with the conceptual framework of MHL introduced by Jorm (2012) [22], which emphasizes recognition, knowledge, and attitudes toward mental health disorders.

The scale consists of 35 items formatted as multiple-choice questions, organized into the following domains: (1) Ability to recognize disorders: Questions Q1–Q8; (2) Knowledge of how to seek information: Questions Q16–Q19; (3) Knowledge of risk factors and causes of mental illness: Questions Q9–Q10; (4) Knowledge of self-treatment: Questions Q11–Q12. (5) Knowledge of professional help available: Questions Q13–Q15; and (6) Attitudes promoting recognition or appropriate help-seeking behavior (stigma): Questions Q20–Q35.

### 2.5. Quality Assurance in Data Collection

To ensure the reliability and validity of the data, several measures were implemented. The survey instrument was pilot tested with a small group of professionals (*n* = 10) to assess clarity, relevance, and technical functionality. Feedback from the pilot test was analyzed, and after discussion within the consortium, no changes were deemed necessary. Additionally, responses were carefully checked for completeness and consistency. Incomplete or inconsistent surveys were excluded from the analysis to maintain data quality.

### 2.6. Data Analysis

#### 2.6.1. Demographic of Participants

To examine the influence of background characteristics on the course outcomes, basic demographic data were analyzed, including work experience, occupation, and participation in previous mental health training programs. A comparison of Mental Health Literacy Scale (MHLS) scores between pre- and post-course respondents was conducted to assess changes in MHL.

#### 2.6.2. Estimation of the Course Effect Size

The primary objective of the analysis was to estimate the effect of the online course on MHLS scores using inverse probability weighting (IPW). While pre- and post-course MHL scores were available, subject-level data for individual participants were not. Instead, the analysis relied on aggregated survey responses from before and after the course.

Due to potential sample imbalances between pre- and post-course respondents, covariates such as working sector, years of experience, and participation in previous courses were included to control for confounding effects. Inverse probability weighting (IPW) was employed to address sample size imbalances by weighing the data based on the probability of course participation. This approach mitigates bias that may arise from differences in characteristics between pre- and post-course respondents [23].

**Figure 1:**
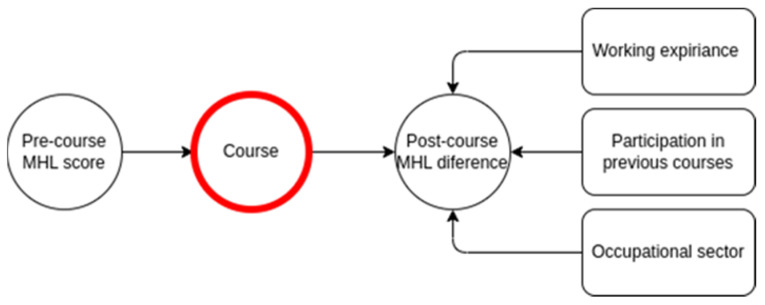
Path diagram of the statistical model.

Data on participants’ country of origin were not collected due to ethical considerations related to anonymity. As a result, stratification by country was not performed, and the country variable was not included in the IPW estimation. Consequently, the data analysis did not aim to assess country-specific effects. A visual representation of the covariate IPW analysis is shown in Figure 1.

##### Frequentist Approach

IPW was conducted in two phases: first, logistic regression was used to model the probability of participation in the post-survey. Covariates such as sector, work experience in years, and participation in previous courses were included. Subsequently, the ‘weights’ were calculated from the inverse of these probabilities. In the second step, the estimated ‘weights’ were used in a linear regression model to determine the difference in MHL scores before and after the course (referred to as the effect of treatment or effect or program effect).

##### Bayesian Approach

A Bayesian model was fitted to estimate the magnitude and direction of the course effect on MHLS scores, assuming non-informative priors. The model was estimated using Markov chain Monte Carlo (MCMC) sampling with 4 chains of 1000 iterations and a warm-up period of 500 iterations. Results were interpreted using the Sequential Effect Existence and Significance Testing (SEXIT) framework, reporting the median of the posterior distribution, its 95% highest density interval (HDI), the probability of direction (pd), and the probability of significance.

The Bayesian approach was chosen to provide a robust estimation of the course effect, particularly in the presence of sample size imbalances and potential confounding factors. It also allows for the incorporation of uncertainty in the estimates, which is particularly useful in studies with smaller post-course sample sizes.

Model convergence and stability were assessed using the R-hat statistic (values < 1.01 indicating convergence) and effective sample size (ESS) (values > 1000 indicating sufficient sampling efficiency) [24,25].

## 3. Results

### 3.1. Demographics of the Participants

The pre-course survey collected responses from 221 participants, with the majority (*n* = 127, 57.5%) completing it in November 2022 (see Supplement Table S1). In contrast, the post-course survey had 58 respondents, most of whom (*n* = 34, 59%) participated in February 2023. The pre-course survey respondents comprised two main cohorts (Supplement Table S2): healthcare professionals (*n* = 120, 54.3%) and educational specialists (*n* = 101, 45.7%).

Analysis of post-course responses (Table 1) revealed a slight increase in the proportion of participants from the healthcare sector (*n* = 39, 67%) compared to the pre-course distribution (*n* = 120, 54%). However, this 12% difference was not statistically significant (*p* > 0.05). Similarly, there were no significant differences between pre- and post-course participants in terms of previous mental health training or work experience. In the pre-course survey, 24% of respondents (*n* = 54) reported prior participation in mental health promotion programs, compared to 19% (*n* = 11) in the post-course survey. Average work experience was also comparable, with 13 (9) years reported in the post-course group and 11 (10) years in the pre-course group.

### 3.2. MHLS Results by Demographical Backgrounds

Participants were categorized by work experience (Table 2): those with <10 years (*n* = 120, mean MHLS = 124.5, SD = 15) and ≥10 years (*n* = 96, mean MHLS = 119.2, SD = 13.8). Both groups showed similar pre-course scores, but the more experienced group demonstrated greater post-course improvement (2.6% vs. 1.8%). Post-course, the <10 years group (*n* = 22) achieved a mean MHLS of 127.3 (SD = 14.70), while the ≥10 years group (*n* = 32) scored 123.3 (SD = 13.6).

Regarding prior training, participants without previous training (*n* = 54) had significantly higher pre-course MHLS scores (129.3, SD = 14.10) compared to those with training (*n* = 167, 119.8, SD = 14.0, *p* < 0.001).

Post-course, untrained participants (*n* = 11) showed greater improvement (4% vs. 1.5%, *p* < 0.05) and higher mean scores (135.7, SD = 9.9) than trained participants (*n* = 47, 122.2, SD = 14.10). By sector, education professionals (*n* = 101) had slightly higher pre-course MHLS scores (120.6, SD = 15.2) than healthcare professionals (*n* = 120, 123.4, SD = 14). Post-course education professionals (*n* = 19) showed greater improvement (3.6% vs. 0.40%) and higher scores (126.0, SD = 12.7) compared to healthcare professionals (*n* = 39, 124, SD = 15.2).

### 3.3. Course Effect on MHLS Score

**Figure 2:**
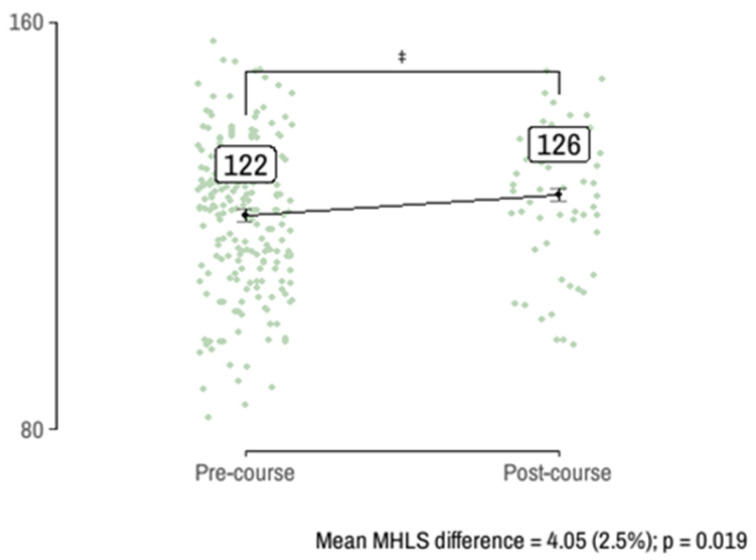
The estimated MHL score in the pre- and post-course survey responses.

Figure 2 illustrates the results of the linear regression model comparing mean mental health literacy (MHL) scores between pre- and post-course groups. The model accounts for class imbalance using inverse probability weighting (IPW) derived from the initial logistic regression step. On average, the course increased MHL scores by 4 points (2.5%), irrespective of professional sector, work experience, or prior training (*p* < 0.05).

**Figure 3:**
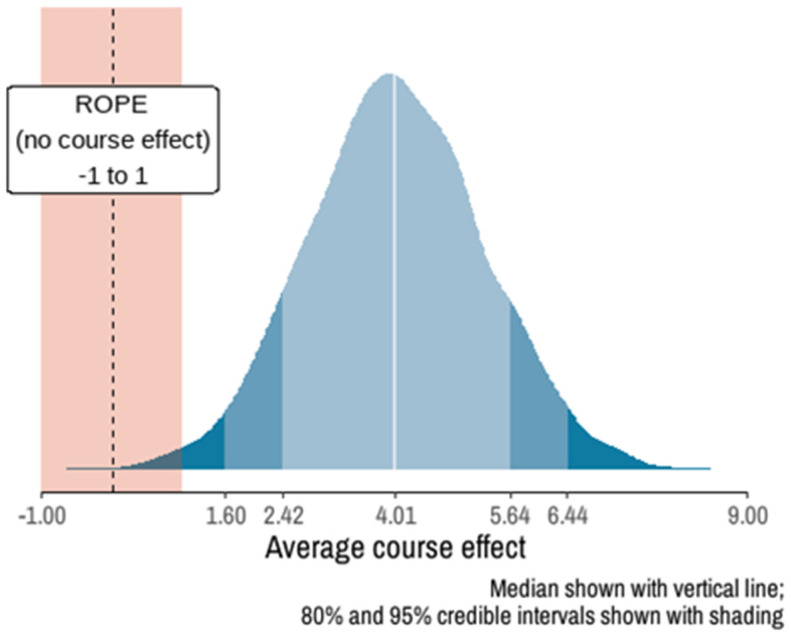
Posterior distribution of the MHL score difference derived from the Bayesian fitted model.

The Bayesian model (Figure 3, Supplement Table S3) estimated an average increase of 4.01 points in MHL scores, with a 95% credible interval of [1.6, 6.44], indicating a significant positive effect. The probability of this effect being greater than zero was 99.92%. The posterior probability distribution showed that there was a 22.62% chance of gaining 3 points, 31.27% for 4 points, and 22.72% for 5 points. The likelihood of achieving an increase of 8 or more points was 2.10%, and the probability of no improvement or decline was minimal (0.13%).

## 4. Discussion

This study aimed to assess the impact of an online course on Mental Health Literacy (MHL) among health, education, and social care professionals working in primary school settings. The findings suggest that the course positively influenced MHL, supporting Jorm’s (1997) [22] definition of MHL as “knowledge and beliefs about mental disorders that aid their recognition, management, and prevention”. These results align with previous research, such as the review by O’Connell, Potel, and Shafran (2021) [26], which found that training courses significantly improved professionals’ knowledge and attitudes toward child mental health.

### 4.1. Impact on Mental Health Literacy Outcomes

The study observed an approximate four-point (2.5%) increase in the Mental Health Literacy Scale (MHLS) scores, with a 99.92% probability that the effect was positive. This improvement is meaningful, as enhanced MHL among school professionals can contribute to better mental health support within school communities.

### 4.2. Influence of Demographic Factors

An analysis of MHLS scores based on demographic characteristics revealed no significant differences between participant groups before and after the course regarding mental health promotion experience or work tenure. However, some trends emerged: professionals with more years of experience and those without prior mental health training showed greater improvement in their MHL scores. Notably, participants with less work experience had higher mean MHLS scores both before and after training, which may be attributed to social factors such as digital literacy, peer support, and evolving health behaviors among younger professionals. The impact of previous training quality remains uncertain, as the study did not differentiate between various training types.

### 4.3. Occupational Background and Training Implications

Post-course, the occupational composition of participants became more homogeneous. While many MHL training programs are designed primarily for teachers [26], effective programs have also been linked to a multimodal or whole-school approach [27]. Some initiatives promoting MHL among children and young people are not exclusively teacher-led [28], in contrast with adult MHL training programs that engage diverse professionals [29]. Despite evidence highlighting the benefits of teacher training, further rigorous research is required to assess the extent to which these programs influence teacher behavior and student mental health outcomes [10,27,30].

A significant portion of participants were nursing students, reinforcing the findings of Hung et al. (2019) [31], who highlighted the experiential benefits of Mental Health First Aid (MHFA) training for nursing students. These results support the integration of MHL training into undergraduate general nursing curricula. Furthermore, the study aligns with Yamaguchi et al. (2021) [32], who suggested that teachers with high MHL are better equipped to identify student mental health challenges and facilitate timely intervention, ultimately fostering confidence in addressing such issues.

### 4.4. Broader Implications and Future Research Needs

This study underscores the critical role of teachers and other school professionals as front-line workers in promoting mental health, preventing mental health problems, and improving MHL [10]. By adopting a holistic approach to MHL [13], training programs for primary school professionals can help reduce stigma and foster positive attitudes towards individuals facing mental health challenges [7,9]. However, as prior studies suggest, improvements in mental health knowledge tend to be more pronounced than shifts in attitudes. This highlights the need for further research, particularly focusing on objective outcome measures and long-term follow-ups to determine the lasting impact of training programs [26].

The findings emphasize the urgent need to address mental health challenges in children and young people, particularly in light of the COVID-19 pandemic and the evolving security situation in Europe and globally. Schools and school professionals play a crucial role in promoting mental well-being, and the results suggest that online MHL training can be an effective means to enhance their competencies. Strengthening the MHL of these frontline workers may contribute to reducing stigma and improving mental health support for children and young people. This study provides promising evidence that structured training programs, such as the one implemented here, can enhance MHL among professionals in primary school settings, ultimately benefiting the wider school community.

## 5. Limitations

This study provides valuable insights into the impact of an online course on mental health literacy (MHL) among health, education, and social care professionals in primary school settings. However, several limitations warrant consideration, which could affect the robustness and generalizability of our findings.

First, the lack of individual identifiers (IDs) due to ethical concerns and data protection prevents the exploration of unobserved individual differences, such as personal attributes, prior knowledge, and unique experiences, which could confound the observed effects of the online course on MHL scores.

Second, the notable limitation is the significant dropout of participants from 221 at the pre-test to 54 at the post-test. This reduction in sample size introduces the potential for selection bias, as the post-test respondents may differ from those who did not complete the post-test. While we cannot confirm that the initial scores of the 54 post-test respondents were lower than 126, we took steps to address this concern by performing a focused analysis (Supplement Figure S1). Specifically, we only compared the top 54 participants based on their MHL scores, both in the pre-course and post-course groups. Even within this more homogenous sub-sample, the Bayesian model still detected a 2.5-point increase in MHLS scores, which suggests that the observed improvement cannot be attributed solely to selection bias. Instead, the increase in scores is likely a reflection of the positive effect of the training program.

Third, the study relies on self-reported data, which introduces the potential for self-reporting bias, including socially desirable responses or inaccurate recall. Additionally, the absence of a control group limits the ability to establish causal relationships between the online course and changes in MHL scores.

Fourth, while the application of the Bayesian approach and inverse probability weighting (IPW) adds sophistication to the analysis, it introduces complexities and assumptions that could impact the accuracy of the results. The Bayesian model’s reliance on non-informative priors may not hold universally, and the use of sample-level data (rather than subject-level data) further limits the analysis.

Fifth, the study’s reliance on English as the language of instruction may introduce selection bias, as participants with better English proficiency are more likely to engage and benefit from the course. This could result in a non-representative sample that does not fully reflect the broader population of professionals in primary school settings.

Sixth, gender distribution among participants in our study was highly imbalanced, with a significantly larger proportion of female participants. Given that MHL levels and attitudes toward mental health can vary by gender, this imbalance may have influenced the results. Future research should investigate gender-related differences in MHL improvement to tailor interventions more effectively. Additionally, ensuring a more balanced gender representation in participant recruitment would enhance the generalizability of findings.

We also acknowledge that, due to the absence of a control group, low post-survey response rates, and lack of participant IDs, stratification based on subgroup characteristics could not be conducted. Such analysis is important for understanding subgroup differences and should be considered in future research. In our study, we employed inverse probability weighting (IPW) to mitigate potential biases from these limitations.

Finally, country-specific estimates and internal reliability by country were not provided, as data on participants’ country of origin were not collected due to ethical considerations related to participant anonymity. This limits the ability to assess potential variations in MHL outcomes across different cultural and national contexts, or to provide country-specific internal reliability data or effect estimates.

## 6. Conclusions

This study evaluated the Well@School online training’s impact on Mental Health Literacy (MHL) among primary school professionals. While results indicate a significant improvement in MHL, there are concerns regarding both internal and external validity due to factors like the lack of a control group, convenience sampling, and self-reported data. The predominance of female participants and language barriers further limit generalizability. Future studies should address these limitations, incorporating randomized controlled trials and considering cultural and linguistic diversity to validate the effectiveness of MHL training across diverse settings.

## Figures and Tables

**Table 1:** A comparison of the observed covariates between study groups (before vs. after survey).

Characteristics	Pre-Course	Post-Course	∆	95% CI	*p*
Healthcare sector	123 (56%)	39 (67%)	12%	−3.2%–26%	0.15
Years of work experience	11 (10)	13 (9)	1.9	−0.86–4.7	0.2
Previous training	54 (24%)	11 (19%)	−5.5%	−18%–7.2%	0.5
Total	221	58			

**Table 2:** The results of MHLS by demographic factors.

		Pre-Course Respondents		Post-Course Respondent		Between Group ∆	
	n	Mean	SD	n	Mean	Mean	SD
Experience							
<10 years	120	124.5	13.8	22	127.3	14.7	2.8
≥10 years	96	119.2	15	32	123.3	13.6	4.1
Sector							
Healthcare	123	123.5	13.8	39	124	15.2	0.5
Educational and Social	98	120.3	15.4	19	126.4	12.7	6.1
Previous training							
No	54	129.3	14.1	11	135.7	9.9	6.4
Yes	167	119.8	14	47	122.2	14.1	2.4

## Data Availability

The data supporting the results of this study are available upon reasonable request from the corresponding author. Due to ethical and privacy restrictions related to participant anonymity, the data cannot be publicly shared. However, aggregated and anonymized data may be provided to interested parties following the necessary ethical approval and confidentiality agreements.

## Supplementary Materials

The following supporting information can be downloaded at: https://www.mdpi.com/article/10.3390/ijerph22030435/s1, Table S1: Survey response over the study period; Table S2: Occupation and sector of the participants; Table S3: Bayesian regression model estimates; Figure S1: Robustness Check of Posterior Bayesian Distributions for Top 54 MHLS Scores: Comparison Between Pre-course and Post-course Groups with Assumed Selection Bias.
