# Supplementary material for: Fostering Mental Health Literacy Among Primary School Professionals: Evaluating the Impact of an Online Training Program ‘Well@School’
Source: Int J Environ Res Public Health. 2025 Mar 15;22(3):435. doi: 10.3390/ijerph22030435 (PMC11942144; doi:10.3390/ijerph22030435)
Supplement: Supplementary file 1 [file ijerph-22-00435-s001.zip › ijerph-3481558-supplementary.pdf]

Supplement Table S1. Survey response over the study period.

	Pre-course respondents		Post-course respondents	
Month	n	%	n	%
2022-10	55	24.9	—	—
2022-11	127	57.5	—	—
2022-12	36	16.3	—	—
2023-01	3	1.4	23	40
2023-02	—	—	34	59
2023-03	—	—	1	2
Total	221	100	58	100

Supplement Table S2. Occupation and sector of the participants

	Pre-course respondents (n =221)		*Post-course respondents (n = 58)*	
occupation	ns	%	n	%
	Educational and Social Professional			
Director	4	4.1	1	5.3
Teacher	86	87.8	18	94.7
Lecturer	2	2.0	—	—
Operations manager	1	1.0	—	—
Social worker	4	4.1	—	—
Social educator	1	1.0	—	—
	98	100	19	100
	Healthcare professional			
Health visitor	24	19.5	2	5.1
Medical doctor	2	1.6	1	2.6
Medical technician	11	8.9	7	17.9
Nurse	38	30.9	19	48.7
Nurse student	19	15.4	6	15.4
Nutritionist	1	0.8	1	2.6
Pharmaceutical technician	2	1.6	1	2.6
Psychologist	11	8.9	2	5.1
Medical laboratory	1	0.8	—	—
Paramedic	1	0.8	—	—
Pharmacy assistant	3	2.4	—	—
Public Health Specialist	6	4.9	—	—
School coach	2	1.6	—	—
School counsellor	1	0.8	—	—
Speech Therapist	1	0.8	—	—
	123	100%	39	100%

Supplement Table S3. Bayesian regression model estimates

Parameter	Median	95 CI (Low)	95 CI (High)	Pd*	Ps**
intercept	122.01	120.29	123.64	1.00	1.00
β (course effect)	4.01	1.60	6.44	0.99	0.99
σ (sigma)	14.12	13.31	15.02	1.00	1.00

*Pd - Probability of direction. ** Ps - Probability of significance.

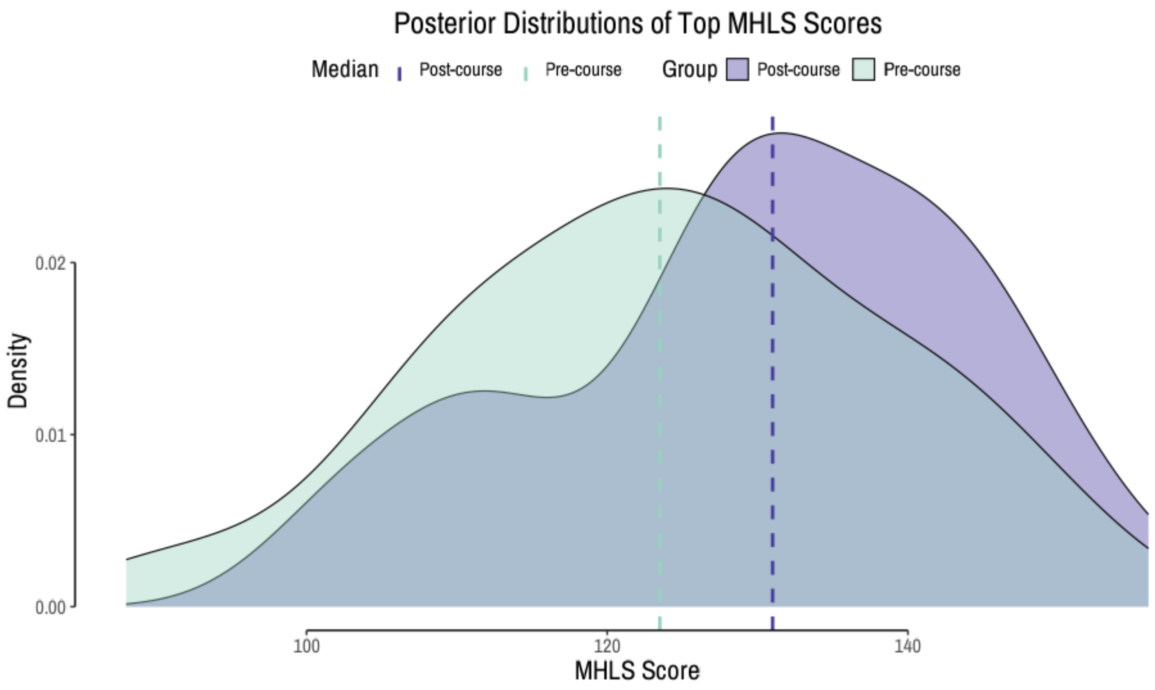

Supplement Figure S1. Robustness Check of Posterior Bayesian Distributions for Top 54 MHLS Scores: Comparison Between Pre-course and Post-course Groups with Assumed Selection Bias.
